# Interdisciplinary Education Model in the Study of Aging: Example and Course Design

**DOI:** 10.1093/geroni/igab046.739

**Published:** 2021-12-17

**Authors:** Yan-Jhu Su

**Affiliations:** University of Massachusetts Boston, Boston, Massachusetts, United States

## Abstract

Collaboration among various disciplines is essential to the gerontology curriculum because it is a a new and comprehensive subject. This presentation will discuss the design of interdisciplinary courses to include practical applications in the study of aging. The presenter will share examples based on personal experience to illustrate how music and psychology may be applied to the study of aging. In addition, the presentation will include analysis of actual course designs to show how different fields can be integrated in the classroom setting. This symposium presentation intends to improve cross-discipline applications as well as help students contribute to and benefit from the study of aging.

